# Molecular Mechanisms Underpinning the Circulation and Cellular Uptake of *Mycobacterium ulcerans* Toxin Mycolactone

**DOI:** 10.3389/fphar.2021.733496

**Published:** 2021-09-16

**Authors:** Bruno Tello Rubio, Florence Bugault, Blandine Baudon, Bertrand Raynal, Sébastien Brûlé, Jean-David Morel, Sarah Saint-Auret, Nicolas Blanchard, Caroline Demangel, Laure Guenin-Macé

**Affiliations:** ^1^ Immunobiology of Infection Unit, INSERM U1221, Institut Pasteur, Paris, France; ^2^ Plateforme de Biophysique Moléculaire, UMR 3528 CNRS, Institut Pasteur, Paris, France; ^3^ CNRS, LIMA, UMR 7042, Université de Haute-Alsace, Université de Strasbourg, Mulhouse, France

**Keywords:** mycolactone, *Mycobacterium ulcerans*, lipid carriers, SR-B1, uptake

## Abstract

Mycolactone is a diffusible lipid toxin produced by *Mycobacterium ulcerans,* the causative agent of Buruli ulcer disease. Altough bacterially derived mycolactone has been shown to traffic from cutaneous foci of infection to the bloodstream, the mechanisms underpinning its access to systemic circulation and import by host cells remain largely unknown. Using biophysical and cell-based approaches, we demonstrate that mycolactone specific association to serum albumin and lipoproteins is necessary for its solubilization and is a major mechanism to regulate its bioavailability. We also demonstrate that Scavenger Receptor (SR)-B1 contributes to the cellular uptake of mycolactone. Overall, we suggest a new mechanism of transport and cell entry, challenging the dogma that the toxin enters host cells via passive diffusion.

## Introduction

Mycolactone is the major virulence factor of *Mycobacterium ulcerans*, the causative agent of a neglected tropical disease called Buruli ulcer (BU) (George et al., 1999). BU manifests as non-healing skin ulcers with massive tissue necrosis surrounding cutaneous infection foci (for review (Guenin-Macé et al., 2019)). Mycolactone is a macrolide composed of a 12-membered ring macrolactone substituted with two polyketide chains that can be isolated from acetone-soluble *M. ulcerans* lipid extracts (George et al., 1998; George et al., 1999). It is responsible for the manifestations of BU disease, including skin necrosis, a relative lack of inflammation and pain at the level of ulcerative lesions, and systemic defects in cellular immune responses (reviewed in (Demangel, 2021)). Mycolactone exerts its immunomodulatory and cytotoxic effects by blocking the Sec61 translocon, a transmembrane channel ensuring the co-translational translocation of nascent secretory proteins into the endoplasmic reticulum (Baron et al., 2016; McKenna et al., 2016; Demangel and High, 2018; Demangel, 2021).

Although bacteria mainly reside in the skin of infected individuals, mycolactone diffuses beyond the sphere of its cytocidal action (Hong et al., 2008; Sarfo et al., 2011; Colucci-Guyon et al., 2020) and gains access to distant tissues. Structurally intact mycolactone is detected in tissues surrounding BU lesions several weeks after completion of antiobiotherapy, suggesting a slow rate of elimination (Sarfo et al., 2011). By grafting a Bodipy fluorophore onto a biologically inert region of mycolactone (Chany et al., 2011; Guenin-Macé et al., 2013; Colucci-Guyon et al., 2020), we have been able to visualize the rapid and body-wide diffusion of intravenously-delivered Bodipy-mycolactone (Bdpy-ML) in zebrafish larvae (Colucci-Guyon et al., 2020). Compared to its isosteric, biologically inactive saturated version (Sat-Bdpy-ML), Bdpy-ML persisted longer in the tissues of injected larvae, suggesting that mycolactone interaction with Sec61 contribute to its retention in host organisms (Colucci-Guyon et al., 2020). Interestingly, we also observed an increased persistence of Bdpy-ML in the vasculature of injected larvae, raising the possibility that a fraction of the toxin stably binds to plasma proteins, thereby delaying clearance.

The lipophilicity and hydrophobicity of mycolactone suggest that its transport in blood plasma is ensured by lipid carrier proteins (Nitenberg et al., 2018; Kubicek-Sutherland et al., 2019). In line with this hypothesis, early attempts to purify *M. ulcerans* toxin showed that its cytotoxicity (George et al., 2000) is retained in both culture filtrates and purified high-density lipoproteins (HDLs) fractions isolated from bacterial cultures (Hockmeyer et al., 1978). More recently, Kubicek-Sutherland and co-workers found that mycolactone forms insoluble aggregates in water or surfactant-free aqueous solutions. Addition of serum or lipoproteins in solution prevented mycolactone precipitation, suggesting that it binds to serum-contained lipid carrier proteins (Kubicek-Sutherland et al., 2019).

At the cellular level, it is currently assumed that extracellular mycolactone reaches its intracellular target Sec61 by passive diffusion through the plasma membrane. This hypothesis is based on observations that fluorescently labeled mycolactone diffused into the cytoplasm of cultured fibroblasts in a non-saturable and non-competitive manner (Snyder and Small, 2003; Chany et al., 2011). However, recent studies using computational simulations and biophysical approaches showed that mycolactone interacts with lipid membrane and alters their architecture (López et al., 2018; Nitenberg et al., 2018). Whether mycolactone is internalized, at least partially, via specialized cell surface receptors remains an open question. Of particular interest are the scavenger receptors (SRs), a family of transmembrane proteins with a wide range of ligands including lipoproteins. Among them, SR-B1 is a widely expressed SR, mainly involved in the bidirectional flux of free cholesterol between cells and HDLs by a non-endocytic mechanism (for review (Shen et al., 2018)). SR-B1 is also known to mediate the selective transport of other lipids, including phospholipids and *α*-tocopherol (Reboul et al., 2006).

In the present study, we combined cutting edge biophysical and cell-based approaches to fully characterize mycolactone’s behavior in solution and to determine the role of different serum lipid carriers in mycolactone bioavailability. Our findings challenge the current dogma that mycolactone enters host cells via passive diffusion by demonstrating the partial contribution of SR-B1 to its cellular uptake.

## Materials and Methods

### Mycolactone and Fluorescent Derivatives

ML was purified from the Malaysian human isolate *M. ulcerans* 1,615, then quantified by spectrophotometry (λmax = 362 nm; log *ε* = 4.29) (Spangenberg and Kishi, 2010) on a V-650 spectrophotometer (JASCO). Bodipy-mycolactone (Bdpy-ML) (λex = 496 nm; λex = 503 nm) was synthesized as previously described (Colucci-Guyon et al., 2020). Stock solutions were prepared in ethanol and diluted at least 1,000x for biophysics or cellular assays. In all cases, controls corresponding to the same volume of vehicle were included.

### Taylor Dispersion of Mycolactone in Ethanol

Taylor dispersion were acquired at 20°C with the Viscosizer TD (Malvern Panalytical Ltd., Worcestershire, United Kingdom). Samples were passed through an uncoated capillary (Malvern Panalytical Ltd., Worcestershire, United Kingdom) with internal and outside diameters of 75 and 360 μm, respectively. UV absorption was monitored at 280 nm. Before sample injection, a stray light correction measurement with L-tryptophan at 11 mg/ml was performed on the system. A sizing measurement with caffeine used as a reference at 1 mg/ml was performed during samples measurements to ensure the good quality of the capillary throughout the experiment.

### Dynamic Light Scattering on Free and Complexed Mycolactone

The presence/absence of precipitates in solution was confirmed by DLS with the DynaPro Plate Reader (Wyatt Technology, United States). This DLS instrument combines an 830 nm laser, a detector positioned at 158°C to collect the signal, and a multi-tau hardware correlator to analyze this signal. A volume of 20 µL of each sample was loaded onto a 384-well (Corning) covered with a transparent film to avoid any evaporation. The plates were centrifuged for 1 min at 3,000 rpm to remove bubbles. Three replicates were performed per well and measurements were conducted at 20°C. A number of 20 acquisitions and 10 s of acquisition time were set up in the Dynamics software. The autocorrelation function was processed using the Dynamics software version 7.1.9 (Wyatt Technology, United States) and converted into size-distribution for analysis.

### Ultracentrifugation—Sedimentation Velocity Experiments

Human serum (10/100 v/v), human albumin (5 mg/ml), human HDL (0.5 mg/ml) or human LDL (0.5 mg/ml) diluted in PBS solution and the subsequent complexes with mycolactone (20 μM) were centrifuged at 42,000 rpm in an Optima AUC analytical ultracentrifuge (Beckman Coulter), at 20°C in an eight-hole AN 50–Ti rotor equipped with 12-mm double-sector aluminum epoxy centerpieces. The concentrations of the individual macromolecules were chosen to be in the range of serum levels and to be detectable by the AUC equipment without saturating the detectors. Detection of the biomolecule concentration as a function of radial position and time was performed by absorbance measurements at 360 nm and by interference detection. At 360 nm, a specific absorbance can be observed for mycolactone in absence of macromolecules’ absorption (Supplementary Figure S1). Ultracentrifugation experiments were performed in PBS (Gibco). Sedimentation velocity data analysis was performed by continuous size distribution analysis c(s) using Sedfit 15.0 software (Brown and Schuck, 2006). All the c(s) distributions were calculated with a fitted fractional ratio f/f0 and a maximum entropy regularization procedure with a confidence level of 0.95. Buffer viscosity and density were calculated from the Sednterp software (www.jphilo.mailway.com/sednterp.htm). Partial specific volumes were also determined with Sednterp.

### Fluorescence Spectroscopy on Plasma

Fluorescence spectra were recorded on human and mouse plasma at room temperature on a JASCO FP-6300 spectrofluorometer equipped with a right angle 10 mm cuvette. The fluorescence was monitored with an excitation wavelength of 490 nm and a maximum emission wavelength between 495 and 550 nm. For human blood measurments, Bdpy-ML was added at 50 or 500 ng/ml to total human blood collected in heparinized tubes, then incubated overnight at 37°C to allow equilibration without inducing cell lysis. A fraction of total blood was used to perform flow cytometry analyses on blood cells. The other fraction was centrifuged 10 min at 2,000 g at RT to collect plasma. To estimate concentrations in samples, serial range dilutions of Bdpy-ML (0.015–1 μg/ml) have been done in human and mouse plasma; areas under the curves obtained from fluorescence data were calculated and a linear regression curve was built between areas and concentrations using Prism software (Supplementary Figure S2).

### Cell Culture and Cellular Assays

Jurkat T cells (E6.1 clone, ECACC#88042803) and THP-1 cells (ECACC#88081201) were cultured in RPMI GlutaMAXTM (Life Technologies), supplemented with 10% heat-inactivated fetal bovine serum (FBS) (Invitrogen), penicillin (100 U/ml) and streptomycin (100 μg/ml). Human primary macrophages were obtained from peripheral blood-derived monocytes isolated by adhesion to tissue culture plastic-ware and cultured with 10 ng/ml human GM-CSF (Peprotech) for 7–12 days. For uptake assays, cells were rinsed in serum-free medium and resuspended in presence of Bdpy-ML (2.5–250 nM) diluted in medium containing either 0.5% or 10% of human serum (Sigma-Aldrich), 0.5% human serum albumin (Sigma-Aldrich), or 0.5 mg/ml human HDL (Sigma-Aldrich) then incubated at 37°C. For release experiments, cells were loaded with Bdpy-ML diluted in medium containing 10% human serum during 1 h at 37°C. Cells were then rinsed twice in serum-free medium before being resuspended in culture medium containing either 0.5% or 10% of human serum (HS), 0.5% human serum albumin (HSA), or 0.5 mg/ml human HDL. In both uptake and release experiments, intracellular content of Bdpy-ML was measured by flow cytometry (FACS) at different time points following addition or removal of Bdpy-ML. Block lipid transfer (BLT)-1, BLT-4, and BLT-5 (Sigma) were used as SR-B1 inhibitors in uptake assays at non-toxic and biologically active concentrations. Zosuquidar and Valspodar (Sigma) were used as MDR1 inhibitors in efflux assays.

### ELISA Assay

Uptake of natural mycolactone was evaluated by measuring the release of IL-2 and TNF-α in Jurkat T cells and THP-1 cells, respectively. Briefly, cells were incubated for 1 h in the presence of 75 ng/ml mycolactone in RPMI medium or in RPMI supplemented with 10% HS, 0.5% HSA (Sigma-Aldrich), or 0.5 mg/ml human HDL, then activated 16 h by addition of PMA (100 ng/ml) and calcimycin (2.5 μg/ml). The release of IL-2 and TNF-α in the culture medium was measured by ELISA using the human IL-2 and TNF-α ELISA Max kits (BioLegend).

### Retroviral Transduction of Sec61α in Primary T Cells

Sec61 wt or R66G Sec61 mutant sequences were cloned upstream of an internal ribosome entry site (IRES) of the pRetroX–IRES-DsRedExpress retroviral vector (Addgene) for simultaneous translation of Sec61α and DsRed in mouse primary T cells as described in (Baron et al., 2016). Platinum-E ecotropic packaging cells (platE, Biolabs) were transduced with pRetroX–IRES-DsRedExpress plasmids (Addgene) containing Sec61α sequences to produce retroviral particles. Mouse CD3^+^ primary T cells were isolated from spleens and lymph nodes by negative selection using the Pan T cell Isolation kit (Miltenyi Biotec) then activated with Dynabeads^®^ Mouse T-activator CD3/CD28 (Miltenyi Biotec) prior to retroviral transduction as described in (Baron et al., 2016). For uptake assay of Bdpy-ML, transduced cells were incubated with different levels of CellTrace (ThermoFisher) to distinguish populations then mixed in equivalent proportion in culture medium before addition of Bdpy-ML for 24 h.

### Western Blot

Cells lysates were prepared in lysis buffer (pH 7.5) containing 20 mM Tris-acetate, 150 mM NaCl, 50 mM NaF, 1 mM EDTA, 1% Triton-X100 and 0.1% protease inhibitors (Sigma-Aldrich). Equal amounts of protein samples were loaded on NuPAGE Bis-Tris gels and transferred to nitrocellulose membranes (Life Technologies). Protein detection was done with anti-actin (#3700, Cell Signaling Technology) and anti-Sec61α (NB120-15575, Novus Biologicals) and revealed with the ECL Prime detection reagent (Cytiva) and chemiluminescence reading on a Fuji LAS-4000 Luminescent Image Analyzer.

### SR-B1 Downregulation by siRNA

siRNA transfections were performed in THP-1 cells using 50 nM of SR-B1 siRNA (On-Target plus human SCARB1, Horizon Discovery) and 1 μl of Lipofectamine RNAimax reagent (Thermofischer) in 500 μl of antibiotic-free medium, following the manufacturer’s instructions. SR-B1 expression was controlled by flow cytometry after 48 h.

### Flow Cytometry

Flow cytometry analysis of THP-1 cells and total blood was performed using the following antibodies. Conjugated anti-human SR-B1 APC (Miltenyi Biotec 130-111-237), CD235a APC Cy7 (BioLegend 3,49,115), CD45 BUV805 (BD Biosciences 6,12,892), CD56 BUV 737 (BD Biosciences 6,12,767), CD11c PeCy7 (BioLegend 3,37,216), CD11b PE (eBioscience 12-0118-41), CD3 PerCP Cy5.5 (BD Biosciences 5,60,835), CD19 BV786 (BioLegend 3,02,239), HLA-DR BUV 395 (BD Biosciences 7,40,302), CD14 BV605 (BioLegend 3,01,834). Labeling of mouse blood was done using the following conjugated antibodies: CD45.2 PE (eBioscience 12-0454–82), CD115 PerCPeF710 (eBioscience 46-1,152-82), CD11b BV650 (eBioscience 48-0112–20), Ly6G BV510 (BD Biosciences 5,63,402), CD3 APC-eF780 (BioLegend 47-0032–82). Fc receptors were blocked using FCR blocking reagent (Myltenyi Biotec). Surface membrane staining was performed in PBS FBS 2%. The Zombie UV fixable viability dye (BioLegend 4,23,107) was used to exclude dead cells. Samples were acquired on a Cytoflex (Beckman Coulter) and analyzed with FlowJo 10 (BD Biosciences).

### Mouse Experiment

Eight-week-old female mice (C57BL/6JRj) were housed and bred under pathogen-free conditions with food and water *ad libitum*. Mice were injected intravenously with 2.5 mg/kg Bdpy-ML first concentrated in ethanol then diluted in physiologic solution (final ethanol concentration 5%). Mice were sacrificed 4, 24, 48, 72, 96 h post-injection (hpi) then 7 days post-injection.

### Statistical Analyses

Statistical analyses and graphical representations were performed using GraphPad Prism software (8.3.1, La Jolla, CA). Unpaired non-parametric Mann-Whitney tests or two-way ANOVA were used as statistical tests.

## Results

### Mycolactone Association With Serum Lipid Carriers Prevents Its Aggregation in Solution

By measuring critical micelle concentrations, Kubicek-Sutherland *et al.* showed that in the 30–60 µM range, mycolactone forms water-insoluble aggregates (Kubicek-Sutherland et al., 2019). Due to this hydrophobic property, mycolactone is highly soluble in ethanol. Indeed, Taylor dispersion analysis showed that solubilized mycolactone has a hydrodynamic radius of 0.48 nm (Supplementary Figure S3A), as expected for well solubilized small components. Dynamic light scattering (DLS) analysis confirmed that mycolactone does not form aggregates in ethanol but forms major aggregates in aqueous solution such as PBS at concentrations >15 nM (Supplementary Figures S3A,B). At a concentration as low as 15 nM in PBS, mycolactone formed particles with radii greater than those of solubilized mycolactone (Supplementary Figure S3). It is therefore likely that mycolactone needs hydrophobic component to be miscible in aqueous buffers. The soluble macromolecules contained in serum that possess hydrophobic patches on their surface or apolar core such as albumin and lipoproteins (Haskard and Li-Chan, 1998; Wasan et al., 2008) represent good candidates to solubilize mycolactone in serum-containing culture media or blood-circulating mycolactone.

**FIGURE 1:**
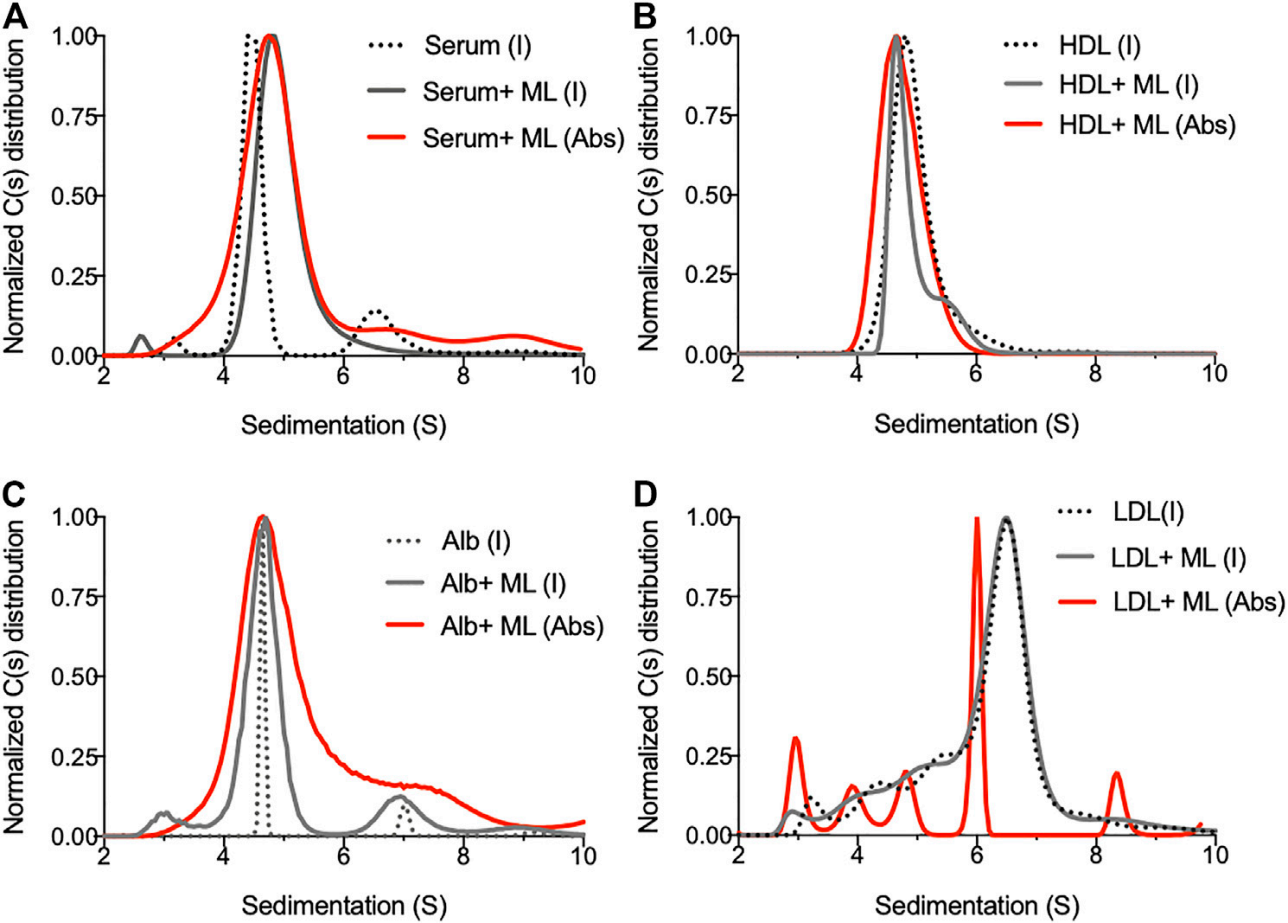
Biophysical analysis of mycolactone behavior in solution. **(A)** Sedimentation profile of species contained in human serum before (dotted line) and after addition of mycolactone (serum + ML, plain line) as detected by interferometry (I, gray line) or absorbance (Abs, red line). **(B–D)** Sedimentation profiles of human HDL (0.5 mg/ml) **(B)** human Albumin (Alb, 5 mg/ml) **(C)** or human LDL (0.5 mg/ml) **(D)** as detected by interferometry (I) or absorbance (Abs) in presence or absence of 20 µM of mycolactone (ML).

In order to identify the biological macromolecules serving as mycolactone carriers in serum, we took advantage of the high separative capacity of analytical ultracentrifugation (AUC). AUC allows the separation of molecular species in solution, based on their shape, size and density and direct analysis through a dual UV/visible and interference detection system, with a resolution that is superior to that offered by other separative technique (Gandhi et al., 2017). Human serum samples spiked with mycolactone and subjected to AUC were analyzed by interference and absorbance, reflecting the sedimentation of proteins and mycolactone, respectively. In the absence of mycolactone, serum contained three main species with sedimentation peaks at 3.2, 4.5 and 6.7 S (Figure 1A). Addition of mycolactone altered the serum sedimentation profile, indicating that molecular interactions occur between mycolactone and serum proteins. Mycolactone detection by absorbance at 360 nm (Spangenberg and Kishi, 2010) revealed that it associates with serum proteins with sedimentation peaks ranging from 3 S to 10 S, particularly at 4.8 S (Figure 1A). We then analyzed the behavior of mycolactone in PBS solutions containing physiological concentrations of macromolecules present in serum: human albumin, HDL or LDL (Figures 1B–D). Interferometry measures showed that mycolactone slightly perturbs HDL sedimentation (Figure 1B), and strongly increases the heterogeneity of albumin species, as illustrated by the broadening of peaks at 4.5 and 6.6 S corresponding to albumin monomer and dimer, respectively, (Figure 1C). Mycolactone detection by absorbance confirmed that it interacts with HDL (4.8 S), as well as albumin monomers and dimers (Figures 1B,C, respectively). When incubated with LDL in PBS, mycolactone induced multiple sedimentation peaks ranging from 3 to 9 S with a main peak at 6 S (Figure 1D), suggesting that it greatly perturbs LDL structural integrity, probably by integrating the apolar core of the LDL. Our data confirm that HDL and LDL improve mycolactone solubility in aqueous solutions (Kubicek-Sutherland et al., 2019), and identify albumin as an additional carrier protein able to solubilize mycolactone in serum.

### Serum Lipid Carriers Impact Bidirectional Transport of Mycolactone Across Cell Membranes

**FIGURE 2:**
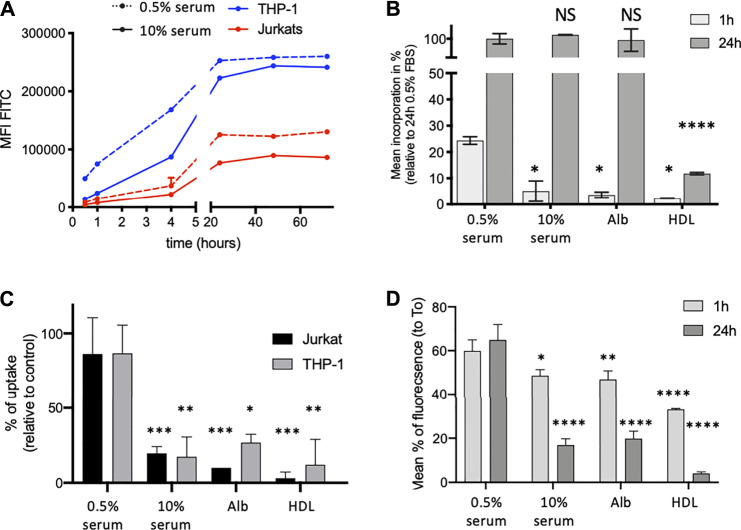
Serum lipid carriers delay intracellular diffusion of Bdpy-ML. **(A)** Time-dependent incorporation of Bdpy-ML (250 nM) as measured by flow cytometry in THP-1 (blue) and Jurkat cells (red) cultured in medium containing 0.5% (dotted lines) or 10% human serum (plain lines). Data are mean fluorescence corrected by FCS of duplicates ± SD and are representative of 3 independent experiments. **(B)** Incorporation of Bdpy-ML diluted in minimal medium (RPMI) ± 0.5% or 10% serum, 0.5% human albumin (Alb) or 0.5 mg/ml human HDL, as measured by flow cytometry in THP-1 cells after 1 (light grey bars) or 24 h (dark grey bars) of incubation. Data are given as percentages of incorporation relative to maximum incorporation (24 h 0.5% serum) and are means of triplicates ±SD. **(C)** Uptake of natural mycolactone by Jurkat cells (black bars) and THP-1 cells (grey bars) after 1 h of incubation with mycolactone (75 ng/ml) in minimal medium (RPMI) ± 0.5% or 10% human serum, 0.5% human albumin (Alb) or 0.5 mg/ml human HDL. Data are expressed as mean percentages of uptake, estimated from a dose-dependent curve of inhibition of IL-2 and TNF-α respectively. Means of duplicates ± SD. **(D)** Intracellular contain of Bdpy-ML in THP-1 cells loaded with Bdpy-ML (T0) then washed and incubated 1 or 24 h in minimal medium (RPMI) ± 0.5 or 10% human serum, 0.5% human albumin (Alb) or 0.5 mg/ml human HDL. Data are given as percentages of retention as compared to T0 and are means of triplicates ± SD. Test 2-way Anova, NS: non significant, *p < 0.1, **p < 0.01, ***p < 0.001, ****p < 0.0001.

We made the hypothesis that mycolactone binding to serum lipid carriers might condition its partition between extra- and intracellular compartments. To examine the potential impact of serum lipid carriers on cellular import and export of mycolactone, we used Bodipy-mycolactone (Bdpy-ML), a previously described fluorescent derivative of mycolactone retaining its biological activity (Chany et al., 2011; Guenin-Macé et al., 2013; Colucci-Guyon et al., 2020). Bdpy-ML associated with serum lipid carriers comparably to natural mycolactone in AUC (Supplementary Figure S4). Using non-differentiated THP-1 and Jurkat cells as models for human monocytes and T cells respectively, we next assessed by flow cytometry the effects of serum lipid carriers on cellular incorporation and release of Bdpy-ML. In both cell types, Bdpy-ML diffused intracellularly in a time-dependent manner, reaching a plateau after 24 h (Figure 2A). Adding 10% serum to the cell culture medium induced a transient delay Bdpy-ML incorporation of by THP-1 cells (Figures 2A,B), an effect that was mimicked by incubation of the cells with albumin at physiological serum concentration (5 mg/ml) (Figure 2B). Notably, physiological concentrations of HDL (0.5 mg/ml) also induced a marked decrease in the early uptake of Bdpy-ML, but in this case, the decrease in Bdpy-ML incorporation persisted after 24 h (Figure 2B). These experiments using Bdpy-ML suggested that HDL, and albumin to a lower extent, may retain mycolactone in the extracellular compartment.

Production of IL-2 by activated T cells, and production of TNF- *α* by THP-1 cells, correlate with the amount of intracellular mycolactone (Supplementary Figure S5). We used this read-out to determine whether extracellular retention of ML by lipid carriers limits its intracellular diffusion. Figure 2C shows that intracellular incorporation of mycolactone, as estimated by its suppressive effect on cytokine production, was decreased by addition of serum and purified lipid carriers, particularly HDL, to the cell culture medium (Figure 2C).

We next examined the impact of serum lipid carriers on the release of intracellular mycolactone in the extracellular medium. To this end, THP-I cells were loaded with Bdpy-ML then rinsed before supplementation of the medium with serum, albumin or HDL. The presence of lipid carriers in the extracellular medium promoted the release of Bdpy-ML from THP-1 cells (Figure 2D). Compared to albumin and serum, HDL displayed a stronger stimulatory effect on Bdpy-ML export by pre-loaded cells (Figure 2D).

Altogether, these data suggested that mycolactone distributes in both the intra-and extracellular compartments, the partition depending on the presence and nature of lipid carriers in the extracellular environment. They supported the view that serum lipid carriers contribute to mycolactone diffusion and persistence *in vivo*.

### In Total Blood, Mycolactone Distributes in Both Plasma and Cell Compartments, With a Preference for Monocytes

**FIGURE 3:**
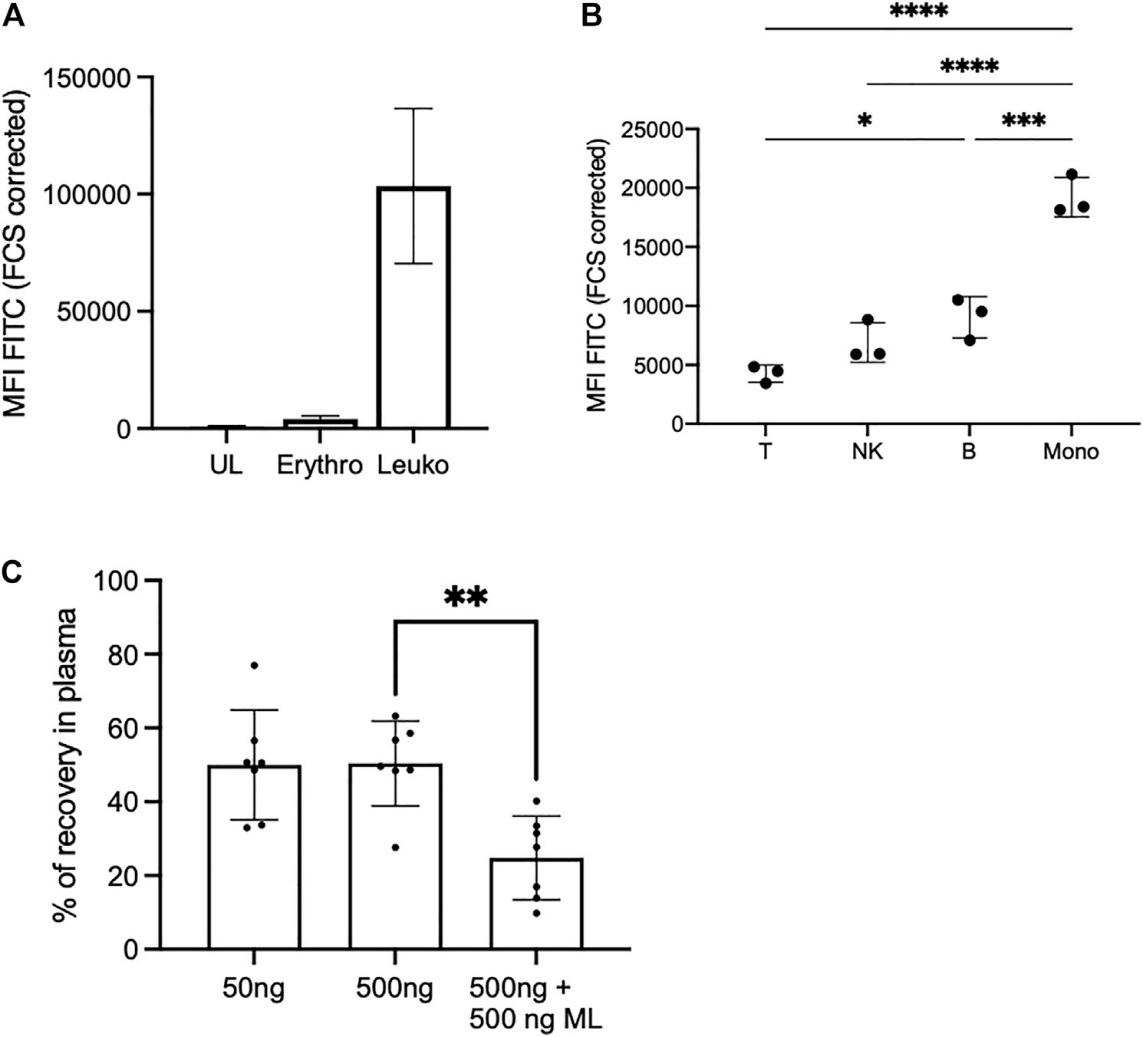
In the blood, Bdpy-ML incorporates differently into cell populations and is largely found in plasma compartment. **(A)** Incorporation of Bdpy-ML as measured by FACS into erythrocytes (Erythro, CD235a+ cells) and leukocytes (Leuko, CD45^+^ cells) after 16 h of incubation in total human blood. Data are mean fluorescence intensities (MFI) ±SD normalized to cell size (FCS corrected) of triplicates. **(B)** Incorporation of Bdpy-ML into CD45^+^ blood cells, including T cells (T), NK cells (NK), B cells (B), and monocytes (Mono), after 16 h of incubation in total human blood. Data are mean fluorescence intensities (MFI) ±SD normalized to cell size of replicates from 3 donors and are representatives of two independent experiments. Test one-way Anova, *p < 0.1, ***p < 0.001, ****p < 0.0001 **(C)** Recovery of Bdpy-ML in plasma fraction as measured by spectrofluorimetry, 16 h after addition of either 50 ng or 500 ng of Bdpy-ML or of 500 ng of Bdpy-ML in presence of 500 ng of natural mycolactone (ML) into total human blood. Data are expressed as percentage of recovery compared to plasma spiked with the same concentrations of Bdpy-ML and are means ± SD of 6 donors.

We previously observed that bacterially-produced mycolactone gains access to serum (Sarfo et al., 2011) and peripheral blood cells (Hong et al., 2008) of infected organisms. Having shown that it associates to lipid carrier proteins *in vitro*, we next asked how mycolactone partitions between cells and serum in human blood, and whether it displays a particular tropism for certain cell subsets. To address this question, Bdpy-ML was added to total human blood before measuring fluorescence levels in cells and plasma (Figure 3). In agreement with our previous studies using radiolabeled mycolactone and mouse blood (Hong et al., 2008), flow cytometric analyses of human blood cell populations revealed efficient internalization of Bdpy-ML by leukocytes (CD45^+^ cells), but not erythrocytes (CD235a+ cells) (Figure 3A). Interestingly, we noticed a differential accumulation of Bdpy-ML across the different CD45^+^ cell subsets, which was independent of the cell size. Monocytes were the most efficient at internalizing Bdpy-ML, followed by B cells, NK cells and T cells (Figure 3B). To quantify Bdpy-ML in plasma, we used the linear relationship between fluorescence emission signals between 495 and 550 nm (λex 490 nm) and Bdpy-ML concentration in the 8–500 ng/ml range (Supplementary Figure S2). In blood incubated with 50 or 500 ng/ml Bdpy-ML, we estimated that 50.4% (±11.5%) of added Bdpy-ML distributed to plasma fractions (Figure 3C). To verify that Bdpy-ML distribution into plasma fractions depended on its mycolactone moiety, we incubated blood samples with 500 ng/ml Bdpy-ML and the same concentration of natural mycolactone. Recovery of Bdpy-ML in plasma decreased by 25% suggesting a competition between Bdpy-ML and mycolactone for association with lipid carriers (Figure 3C). We concluded that mycolactone distributes to both plasma and human blood cells, with a preference for monocytes.

### Transport and Persistence of IV-Delivered Mycolactone in Blood Cell and Plasma

**FIGURE 4:**
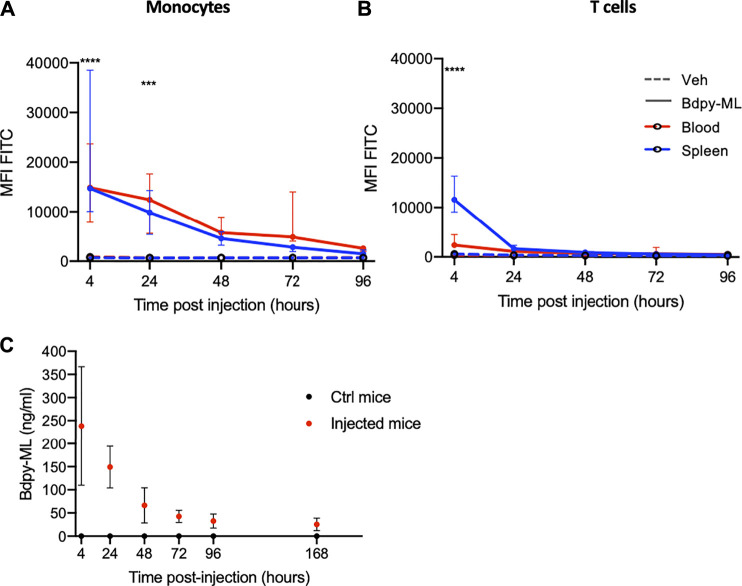
In the mouse, intra-venously delivered Bdpy-ML is detected in plasma up to 7 days post-injection. **(A, B)** Bdpy-ML incorporation, as measured by FACS, in blood (red) or spleen (blue) monocytes **(A)** and CD3^+^ T cells **(B)** in mice injected with either Bdpy-ML (plain lines) or with vehicle (dotted lines). Data are presented as mean fluorescence intensities (MFI) across time and are median values ±SD, at least 3 mice per group. **(C)** Estimated concentration of circulating Bdpy-ML (from standard curve) in plasma following intravenous injection. Data are median values ±SD, at least 3 mice per group. Test 2-way Anova, ***p < 0.001, ****p < 0.0001.

We used the mouse model to explore further mycolactone distribution and persistence *in vivo*. Mice were injected intravenously with Bdpy-ML (2.5 mg/kg) and fluorescence levels were measured in blood cells and plasma, as well as splenocytes, at several time points post-injection using the above-described approaches. In agreement with our *in vitro* data (Figure 3B), Bdpy-ML was relatively better internalized by monocytes than T cells, 4 h post-injection (hpi) (Figures 4A,B). After 24 h, Bdpy-ML was almost cleared from T cells (Figure 4B) while persisting up to 72 h in monocytes (Figure 4A). Profiles of Bdpy-ML incorporation were very similar in cells isolated from blood and spleen after 4 hpi, suggesting an efficient distribution of blood-contained Bdpy-ML to this organ. We estimated plasma concentrations of circulating Bdpy-ML with the approach described in Figure 3, and a standard curve made with serial dilutions of Bdpy-ML in mouse plasma (Supplementary Figure S6). At 4 hpi, plasma levels of Bdpy-ML were highly variable with mean values estimated at 238 ng/ml ± 128 (Figure 4C), which corresponded to 0.78% of injected Bdpy-ML (Mean mouse weight: 18.4 mg ± 0.6, estimated blood volume 77–80 ml/kg (Mitruka and Rawnsley, 1981)). Plasma concentrations of Bdpy-ML then decreased overtime to stabilize at 42 ng/ml ± 13 after 72 h. Notably, Bdpy-ML was still detected 7 days post-injection (Figure 4C). In line with our findings in the zebrafish (Colucci-Guyon et al., 2020), data obtained in intravenously injected mice support a rapid and almost complete diffusion of blood contained mycolactone into body organs. The long persistence of low levels of Bdpy-ML in plasma is consistent with a slow release of tissue-contained mycolactone in the blood compartment, where our *in vitro* data suggest that it largely localizes in the plasma.

### The Differential Incorporation of Bdpy-ML Is Not due to ML Binding to Sec61

**FIGURE 5:**
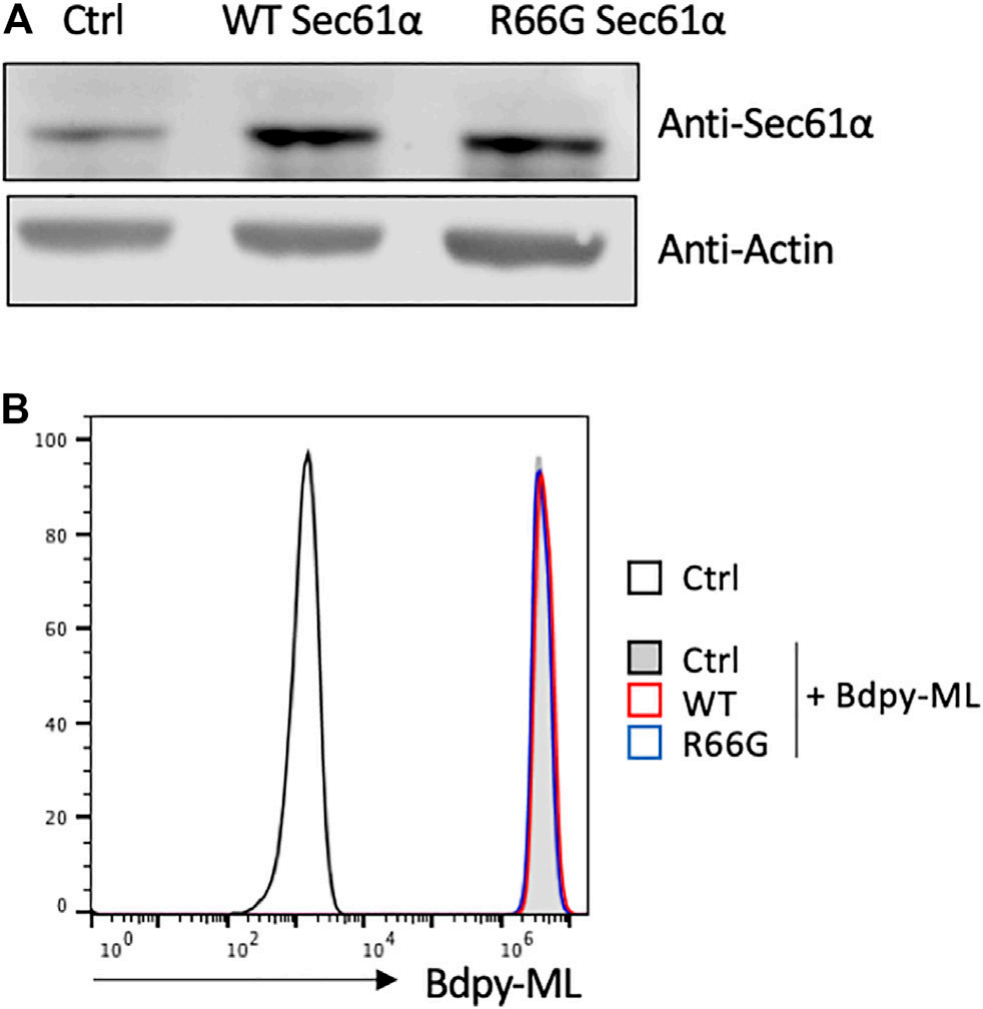
Intracellular incorporation of Bdpy-ML in lymphocytes is independent of the level of Sec61α. **(A)** Detection, as done by western blot, of Sec61α (Top lane) or Actin (bottom lane) in primary mouse T cells either non-transfected (Ctrl) or transfected with the wild type form of Sec61α (WT), or with the mycolactone-resistant Sec61 carrying the R66G mutation (R66G) **(B)** Histogram showing incorporation of Bdpy-ML as measured by FACS in non-transfected mouse T cells (Ctrl) or in WT- or R66G-transfected cells. Cells were loaded with different doses of cell trace to identify each population, mixed in equivalent proportion and incubated 24 h with Bdpy-ML. Incorporation of Bdpy-ML was measured by FACS in each cell population identified by cell trace marker.

Our *in vitro* and *in vivo* data revealed a differential incorporation of Bdpy-ML by monocytes and lymphocytes. We hypothesized that this difference could result from a relatively higher concentration of Sec61 in monocytes. To test this hypothesis, we compared the incorporation of Bdpy-ML in Jurkat T cells transfected with either the wild type (WT Sec61α) or the mycolactone-blind R66G mutant of Sec61α (Baron et al., 2016). We previously demonstrated that over-expression of R66G Sec61α rescues the cytokine responses of mycolactone-treated T cells (Baron et al., 2016). Compared to untransfected cells, transfected cells showed higher and comparable expression of the WT or the mutant form of Sec6α as (Figure 5A). Bdpy-ML uptake was comparable in untransfected cells and cells over-expressing WT or R66G Sec61α (Figure 5B), suggesting that mycolactone uptake is independent of Sec61α level.

### The Scavenger Receptors SR-B1 Partly Contributes to Intracellular Mycolactone’s Uptake

**FIGURE 6:**
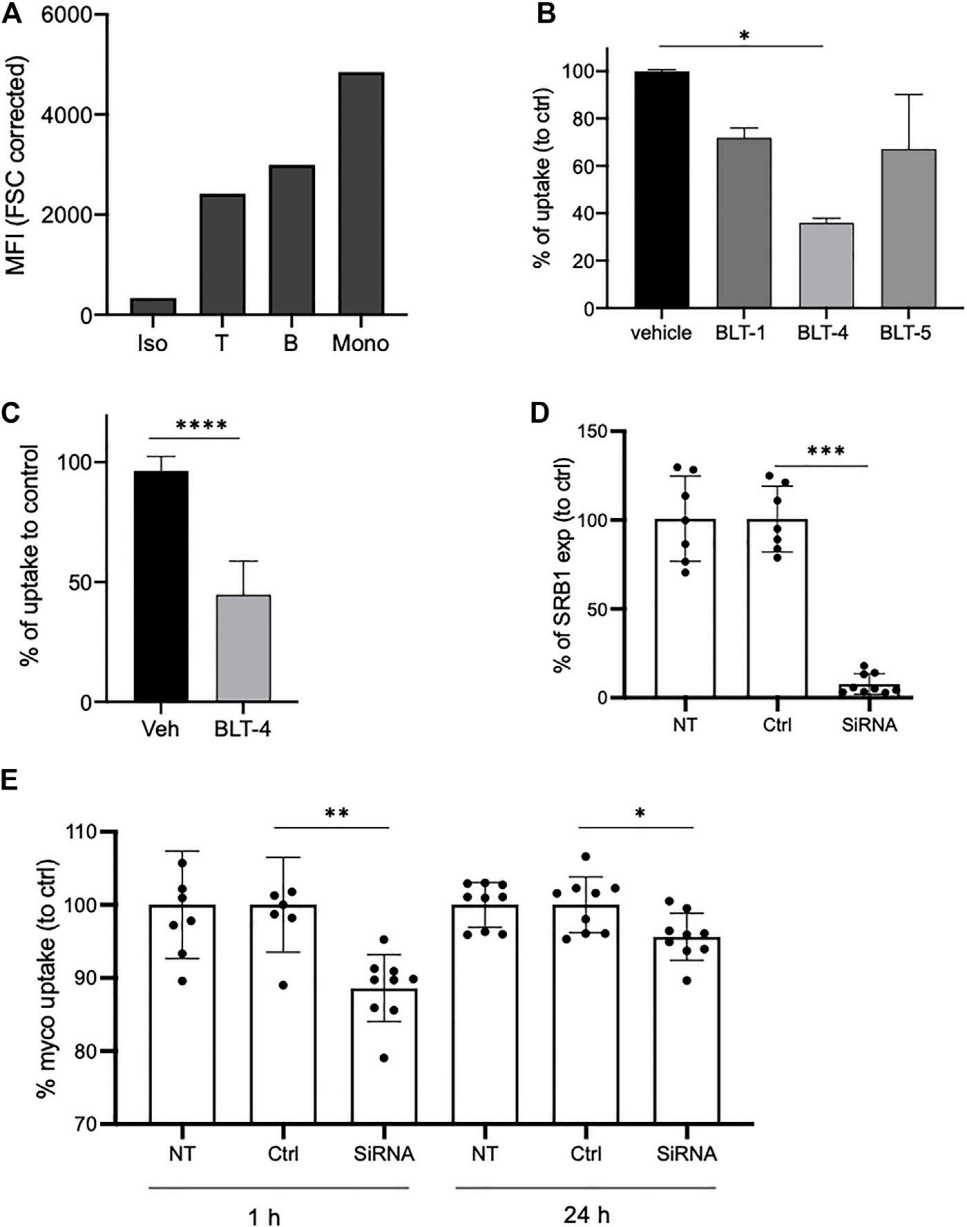
SR-B1 partly contributes to uptake of mycolactone in monocytes. **(A)** SR-B1 expression as measured by flow cytometry in human blood T cells (T), B cells or monocytes (Mono) compared to isotype labeling (Iso). Data are shown as mean fluorescence intensities (MFI) normalized to cell size (FCS corrected). **(B)** Incorporation of Bdpy-ML into purified human primary monocytes after 1 h in presence of vehicle or of the BLT inhibitors: BLT-1 (10 µM), BLT-4 (75 µM), and BLT-5 (150 µM). Data are shown as percentage of uptake to control (vehicle) and are means ± SD of triplicates. Test one-way Anova, *p < 0.1. **(C)** Incorporation of Bdpy-ML into undifferentiated THP-1 cells after 1 h in presence of vehicle (Veh) or BLT-4 (75 µM). Gate on viable cells. Data are shown as percentage of uptake to control (Veh) and are means ± SD of 3 independent experiments. Test Mann-Whitney, ****p < 0.0001. **(D)** SR-B1 expression in non-transduced cells (UT), cells transduced with non-targeting siRNA (Ctrl) or with SR-B1 siRNA (siRNA). Data are expressed as percentage to control and are means ± SD of 3 independent experiments with at least 2 replicates per group. **(E)** Bdpy-ML uptake in non-transduced (NT) or transduced THP-1 cells with non-targeting siRNA (Ctrl) or with SR-B1 siRNA (siRNA), after 1 or 24 h of incubation with Bdpy-ML. Data are means ± SD of 3 independent experiments with at least 2 replicates per group, expressed as percentage to control. Test Mann-Whitney, *p < 0.1, **p < 0.01, ***p < 0.001.

Addition of equimolar or higher concentrations of natural mycolactone induced a slight yet significant decrease in Bdpy-ML uptake by THP-1 cells (Supplementary Figure S7), suggesting that mycolactone uptake does not rely exclusively on passive diffusion. The scavenger receptor SR-B1 is an HDL receptor whose primary role is to selectively take up HDL-derived cholesteryl esters into cells and tissues by a non-endocytic mechanism. Since mycolactone associates with HDL, we hypothesized that SR-B1 may contribute to its cellular import. In support of this view, blood cell subsets incorporating the highest levels of Bdpy-ML (Figure 3) were those expressing the highest levels of SR-B1 (Figure 6A). To assess SR-B1 contribution to cellular import of mycolactone, we used a panel of pharmacological inhibitors (BLT-1, BLT-4, and 5) that specifically block SR-B1-dependent lipid transport from HDL (Nieland et al., 2002; Nieland et al., 2008). We measured the uptake of Bdpy-ML into primary human macrophages after 1 h of incubation. In the presence of the inhibitors, Bdpy-ML uptake showed variable and partial inhibition, with BLT-4 having the greatest effect (Figure 6B). BLT-4 also significantly reduced mycolactone uptake by THP-1 cells after 1 h (Figure 6C).

To test the contribution of scavenger receptors SR-B1 to mycolactone uptake by an independent approach, THP-1 cells were transfected with anti-SR-B1 or unrelated siRNAs. Forty-eight hours post-transfection, SR-B1 expression was down-regulated in 72% of cells (Supplementary Figure S8). In these cells, anti-SR-B1 siRNAs reduced SR-B1 expression by 92% (±5%) (Figure 6D). We then compared cells transfected with anti-SR-B1 or unrelated siRNAs for Bdpy-ML incorporation after 1 and 24 h (Figure 6E). Compared to control cells and cells transfected with control siRNAs, cells transfected with anti-SR-B1 siRNAs had a 10% reduction in ability to internalize Bdpy-ML after 1h, which persisted to a lesser extent after 24 h (Figure 6E). Collectively, the data reported in Figure 6 suggest that SR-B1 contributes to the cellular uptake of mycolactone.

## Discussion

Here, we used a combination of biophysical and cellular approaches to characterize mycolactone’s behavior in solution as well as mechanisms of cellular uptake. Our data obtained *in vitro* strongly support the view that mycolactone cannot circulate as free solubilized macromolecule in the aqueous environment of the circulatory system but rather associated to plasma lipid carriers.

We show that in human blood, a large proportion of mycolactone is delivered to the plasma fraction, in association with serum lipid carriers including albumin, HDL and LDL. This result differs quantitatively from our previous study using mouse blood and natural mycolactone, where the proportion of mycolactone distributing to the extracellular fraction was considered marginal. At that time, quantitative detection of mycolactone in plasma was performed by ESI-LC-MS analysis of acetone-soluble lipid extracts (Hong et al., 2008), an approach later found to be poorly efficient for recovery of serum associated mycolactone (Sarfo et al., 2011).

Our recent work performed in zebrafish (Colucci-Guyon et al., 2020) had shown that Bdpy-ML diffuses rapidly from the blood compartment toward most tissues. The present study confirms this observation and demonstrates that a part of intravenously-delivered Bdpy-ML persists in mouse plasma for at least 7 days. It supports the notion that serum lipid carriers play a role as transporters and reservoirs of mycolactone in BU patients. This notion is consistent with the body-wide distribution and persistence of mycolactone *in vivo* (Sarfo et al., 2011; Colucci-Guyon et al., 2020). Indeed, albumin distributes into the extravascular spaces of all tissues (for review (5 Human Albumin, 2009)) and its long half-life confers albumin-bound drugs elevated persistence (Larsen et al., 2016). We propose a distribution model where bacterially-produced mycolactone gains access to the peripheral blood to rapidly diffuse into the tissues of infected organisms, and diffuses back slowly from these tissues into the blood compartment. While low, plasmatic levels of mycolactone in BU patients may be relevant indicators of mycolactone persistence in treated patients.

Albumin, HDL and LDL have in common the presence of apolar regions forming hydrophobic interactions (Wasan et al., 2008). As the most abundant protein in the plasma, albumin likely represents the major carrier of mycolactone in patient blood. Previous studies have demonstrated that albumin contains two hydrophobic patches at its C and N-terminal end able to bind hydrophobic compounds (Haskard and Li-Chan, 1998). In addition, albumin efficiently transports fatty acids (FA) and has about 7 FA binding sites with variable affinity (van der Vusse, 2009). We observed that FA-free albumin is superior to FA-bound albumin for retention of mycolactone in the extracellular compartment (Supplementary Figure S9), suggesting that mycolactone can bind to albumin at the same sites as FA. Our *in vitro* and cellular studies also highlighted the potential importance of HDL as mycolactone transporter and reservoir. HDL present a central hydrophobic core mostly composed of nonpolar lipids and a polar monolayer of phospholipids, free cholesterol and apolipoproteins, a structure that is appropriate to the transport of lipophilic compounds (Lund-Katz et al., 2010; Lacko et al., 2015). Depending on their polarity and hydrophobicity, lipophilic molecules transported by HDL preferentially localize within the core or the external layer (Wasan et al., 2008). Our results suggest that mycolactone can form interactions with one or the other of these HDL constituents. Of note, recent studies using artificial monolayers and computational modeling indicated that mycolactone can directly interact with lipid membranes (López et al., 2018; Nitenberg et al., 2018; Aydin et al., 2019). Whether mycolactone stably associates with plasma membranes *in vivo*, and how it partitions within the various cell compartments remains unknown.

We found that interactions between mycolactone and lipid carriers determine mycolactone’s bioavailability for peripheral blood cells. When mycolactone gains access to the systemic circulation, reversible binding occurs with plasma proteins. Association/dissociation kinetics of drug binding to plasma carriers determine their biodistribution, those with a high capacity to bind plasma proteins being less able to reach perfused organs. Likewise, mycolactone association with plasma proteins should promote its persistence in the blood circulation.

The mechanisms underpinning mycolactone intracellular diffusion are largely unknown. The diffusion kinetics of a fluorescent derivative of mycolactone and the lack of effect of inhibitors of major signaling pathways suggested a non-endocytic mechanism of diffusion (Snyder and Small, 2003). As suggested by recent studies using computational simulations and model lipid membranes, mycolactone may have ability to cross plasma membranes directly (Nitenberg et al., 2018; López et al., 2018; Nieland et al., 2004). Our observations that mycolactone associates with albumin and lipoproteins suggest that other receptor-mediated mechanisms of mycolactone import occur. In support of this view, we observed an elevated uptake of mycolactone by the monocytic subset of peripheral blood cells, which expresses higher levels of the HDL receptor SR-B1 (Figure 6A). Our siRNA knock-down of SR-B1 led to a moderate but significant reduction of mycolactone uptake by monocytes, showing that SR-B1 is involved. Based on this result, we propose that variations of SR-BI expression may contribute to the variable uptake of mycolactone across cell populations. How SR-B1 transports mycolactone remains to be clarified but the results with BLT inhibitors suggest a mechanism of import differing from that of cholesterol. Indeed, the most potent inhibitor of cholesterol uptake (BLT-1) had little impact on mycolactone uptake, while BLT-4, which cross inhibits both SR-B1 and the ATP binding cassette (ABC) transporter ABCA1 (Nieland et al., 2004), had a relatively higher effect. ABCA1 is involved in cholesterol and phospholipid efflux from cells to Apolipoprotein A1 (ApoA-1) to generate HDL (Vedhachalam et al., 2007). In addition to this well-established role, ABCA1 has been proposed to facilitate bidirectional sterol flux through the plasma membrane (Yamauchi et al., 2015). Although its contribution to mycolactone is quantitatively modest, SR-B1 provides the first example of membrane transporter contributing to mycolactone import into host cells. In addition to this mechanism, albumin and/or lipoproteins membrane receptors other than SR-B1 could mediate mycolactone uptake by host cells, by transfer of mycolactone from carriers to plasma membrane or by endocytosis of mycolactone-carrier complexes. Notably, it will be interesting to investigate the contribution of the broadly-expressed neonatal Fc receptor (FcRn) that plays a key role in homeostatic regulation of albumin (Sand et al., 2015).

In addition to a mechanism of import, we investigated a potential mechanism of mycolactone detoxification through the multidrug resistance protein 1 (MDR1 also known as ABCB1 or P-glycoprotein), an ABC transporter. MDR1 is an efflux pump known to export various amphiphilic compounds including drugs and lipids and to reduce the accumulation of xenobiotics in the cell (Bossennec et al., 2018). To examine a possible contribution of MDR1 in mycolactone efflux, we monitored the decrease of intracellular fluorescence in THP-1 cells loaded with Bdpy-ML, in the presence of Valspodar or Zosuquidar, two specific and highly efficient inhibitors of MDR1 (Bossennec et al., 2018) (Supplementary Figure S10). Under the conditions tested, we did not observe any impact of the inhibitors on the release of Bdpy-ML, ruling out a possible involvement of MDR1 in mycolactone efflux.

Beyond the scope of BU disease, we previously demonstrated the therapeutic potential of mycolactone to treat inflammatory disorders in mouse models (Guenin-Macé et al., 2015). A better knowledge of mycolactone interaction with serum carriers is thus essential for the therapeutic use of mycolactone. Many studies report efforts to develop drug delivery systems using albumin or HDLs as a drug carriers (Wasan et al., 2008; Larsen et al., 2016; Hoogenboezem and Duvall, 2018; Tayyab et al., 2021). Accumulation of albumin in solid tumors as well as overexpression of SR-B1 in most malignancies warrant developing carrier-based drug delivery systems for tumor targeting (Larsen et al., 2016; Raut et al., 2018). The combination of mycolactone with albumin and HDLs looks attractive for the therapeutic use of mycolactone as it could promote its delivery toward specific cells or tissues with high affinity for these carriers such as tumors.

In conclusion, our findings reveal the importance of serum lipid carriers in mycolactone biodistribution, at the organism and cellular levels. Here we demonstrate that albumin and lipoproteins act as natural drug delivery systems for mycolactone, having consequence on its biological activity and probably on its pharmacokinetics. Our findings open new perspectives for the monitoring of BU patients, based on the measurement of plasma levels of mycolactone as well as for the use of mycolactone-bas557) ed therapeutics.

## Data Availability

The original contributions presented in the study are included in the article/Supplementary Material, further inquiries can be directed to the corresponding author.
