# Supplementary material for: Molecular Mechanisms Underpinning the Circulation and Cellular Uptake of Mycobacterium ulcerans Toxin Mycolactone
Source: Front Pharmacol. 2021 Sep 16;12:733496. doi: 10.3389/fphar.2021.733496 (PMC8481864; doi:10.3389/fphar.2021.733496)
Supplement: Supplementary file 1 [file DataSheet1.PDF]

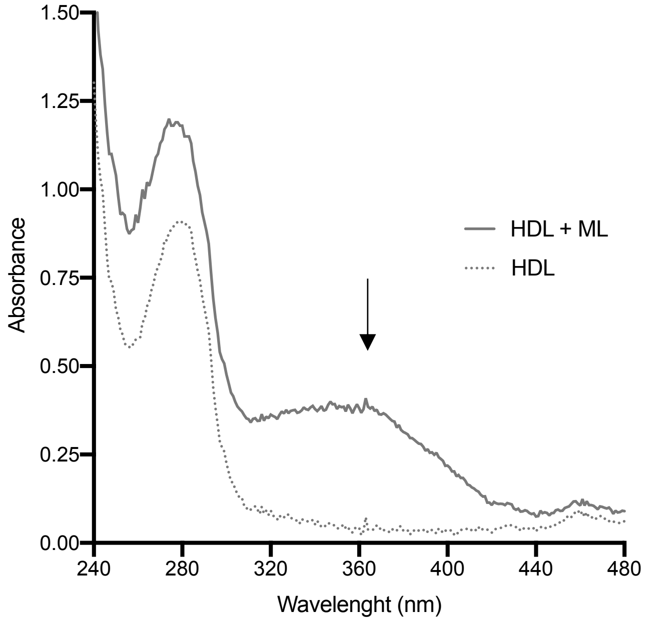

Figure S1. Absorbance spectrum of PBS containing either HDL (dotted line) or HDL + mycolactone (plain line). Specific absorbance at 360nm can be observed in presence of mycolactone (arrow).

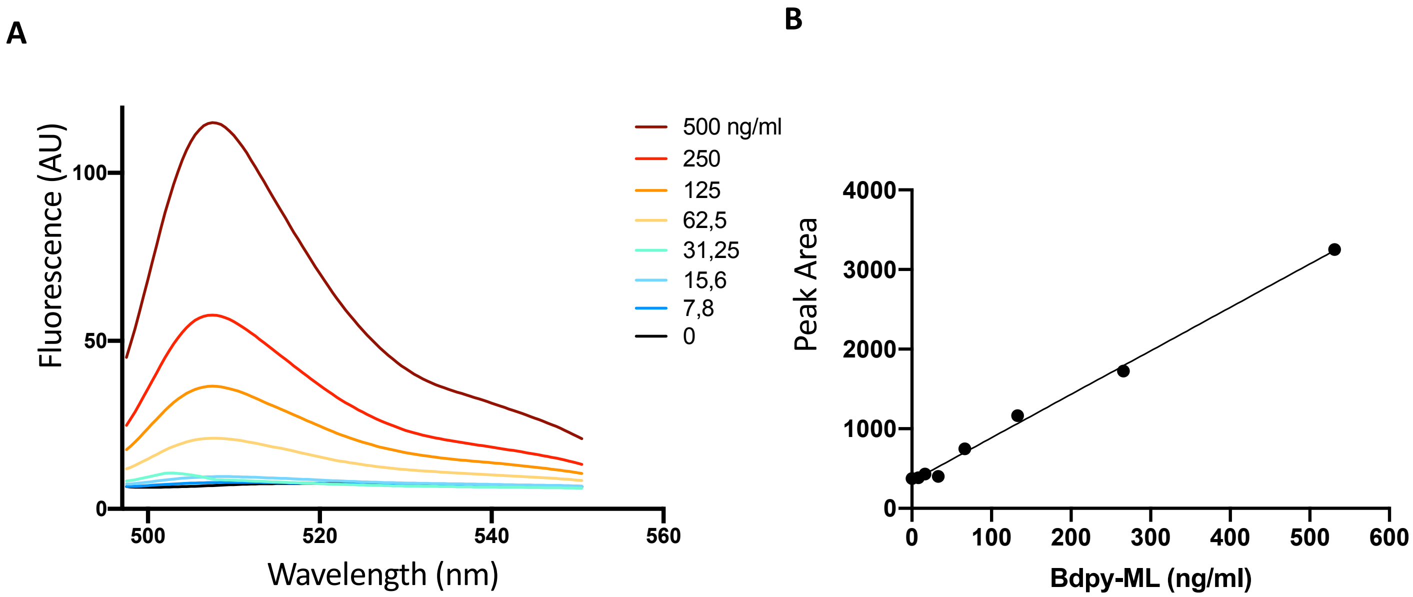

Figure S2: Detection by spectrofluorimetry of Bdpy-ML diluted in human plasma
(A) Fluorescence intensity of serial dilutions of Bdpy-ML (0-500ng/ml) in human plasma as measured by spectrofluorimetry (λex 490, λem 495-550). (B) Linear regression between area under curves (Peak area) obtained by spectrofluorimetry and concentration of Bdpy-ML in human plasma in the 8-500 ng/ml range.

A

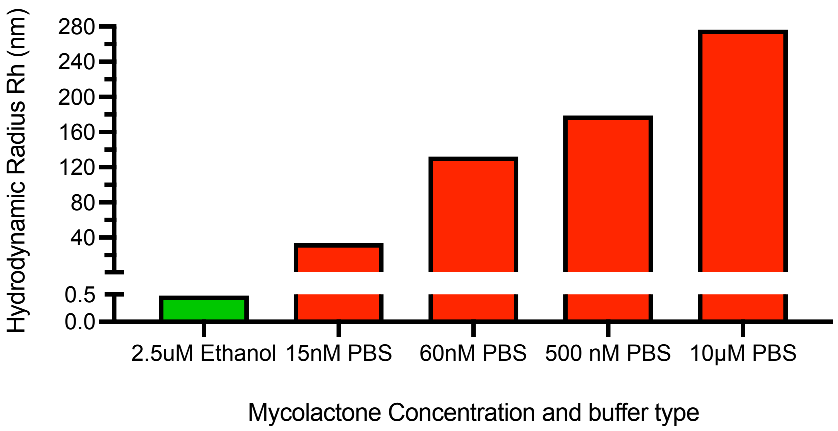

B

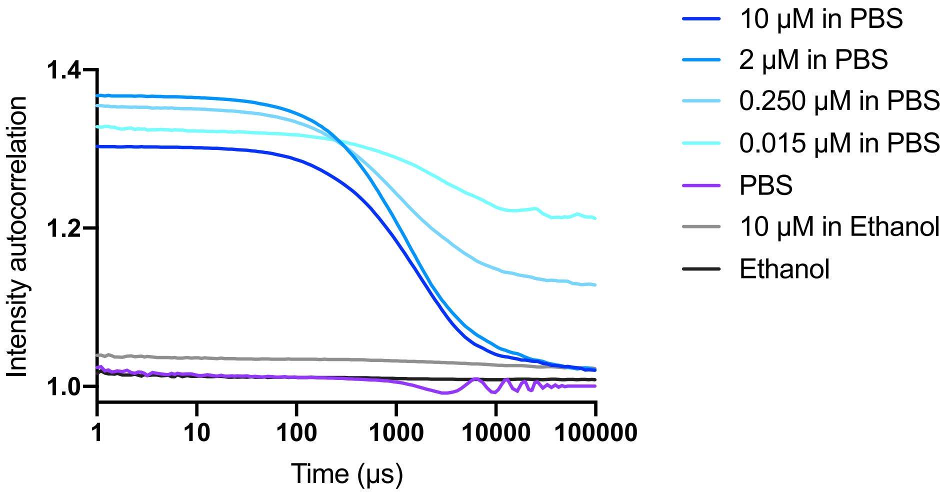

Figure S3: Solubilization of mycolactone. (A) Average hydrodynamic radius of mycolactone determined by Taylor dispersion and DLS in green and red, respectively. Mycolactone solubilized in PBS shows the presence of large aggregates, more than 100 times bigger than the well solubilized mycolactone in Ethanol as detected by taylor disersion. **(B)**. Dynamic light scattering autcorrelation curves for filtered PBS, ethanol, mycolactone solubilized in ethanol (10 μM), and in PBS (15 nM, 250nM, 2μM, 10μM). The mycolactone solubilized in Ethanol is soluble and no aggregates can be detected as shown by the low amplitude of the autocorrelation curve similar to buffer (PBS and ethanol). The mycolactone solubilized in PBS shows aggregates at all concentration tested with high amplitude of the autocorrelation curve.

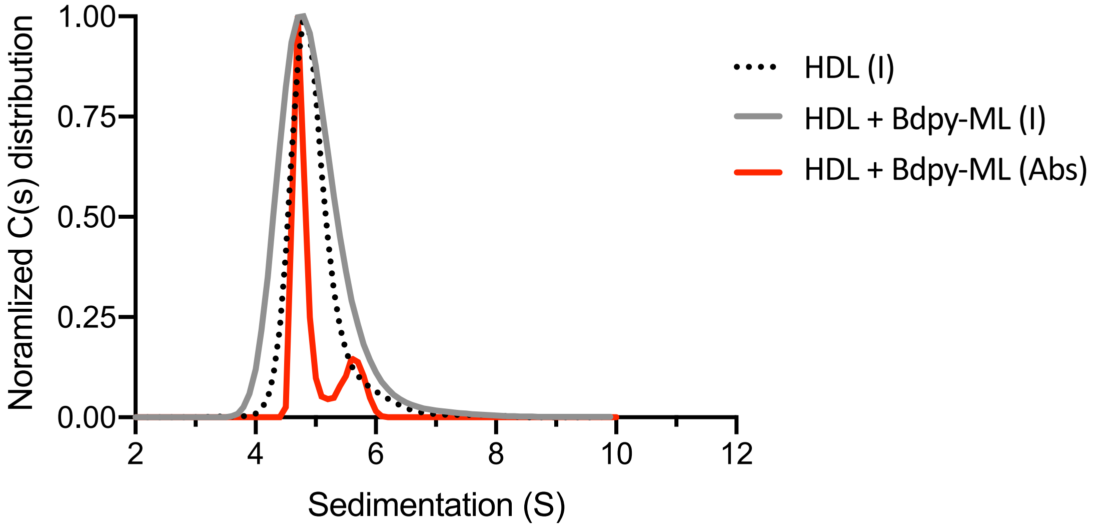

Figure S4. Analysis of sedimentation of human HDL in PBS solution in presence or absence of Bdpy-ML as detected by interferometry (I). Bdpy-ML detection by Absorbance (Abs) shows that Bdpy-ML interacts with HDL species.

A **B**

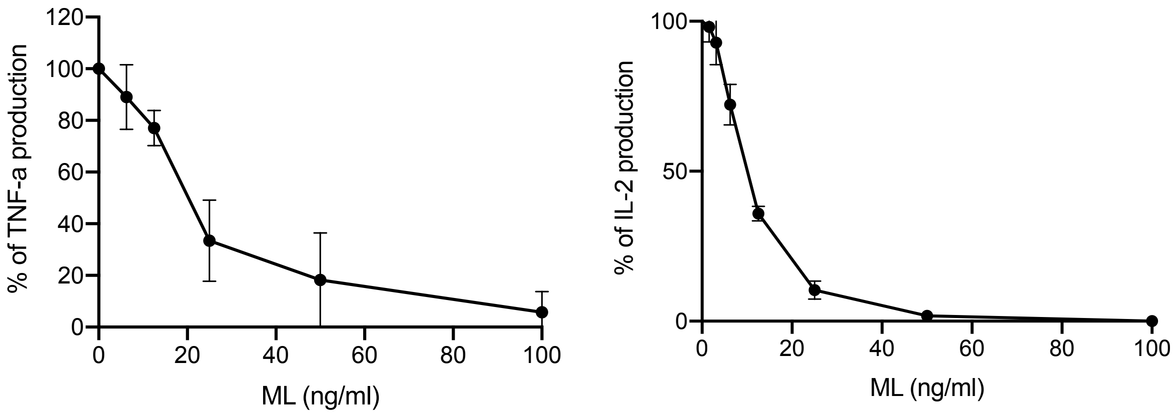

Figure S5. Mycolactone-induced dose-dependent inhibition of cytokines production. Production of TNF-α by activated THP-1 (A) and IL-2 by Jurkat cells (B) in presence of increasing doses of mycolactone (ML) as measured by ELISA. Data are expressed as percentage of production to control (DMSO) and are means values of duplicate.

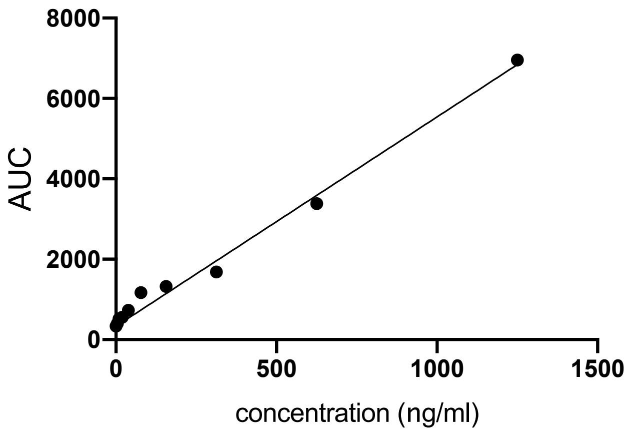

Figure S6: Standard curve of serial dilutions of Bdpy-ML in mouse plasma
Standard curve build from serial dilution of Bdpy-ML in mouse plasma and corresponding area under curve (AUC) of emission peaks measured by spectrofluorimetry (λex 490, λem 495-550). Linear regression is obtained for concentrations of Bdpy-ML in the 5-1200 ng/ml range.

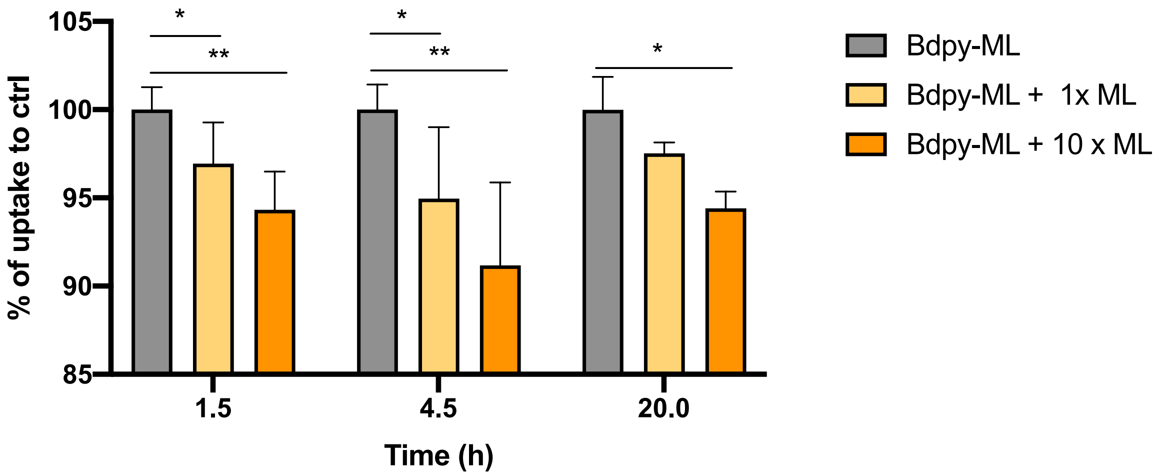

Figure S7. Uptake of Bdpy-ML in presence of equimolar or 10x excess mycolactone (ML) in THP-1. Fluorescence levels as measured by flow cytometry, reflecting Bdpy-ML incorporation in THP-1 in presence or absence (gray bars) of equimolar concentration of mycolactone (light orange) or 10x excess concentration of mycolactone (orange bars). Data are mean percentages of uptake +/- SD compared to Bdpy-ML condition for each time point and are pooled data from 3 independent experiments in triplicates.

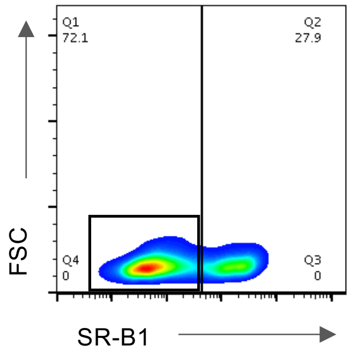

Figure S8: Dual parameter density plot of THP-1 cells, 48h post-transduction with SR-B1 siRNA, showing decreased expression of SRB1 in 72% of cells. Black rectangle indicates gating on transfected cells.

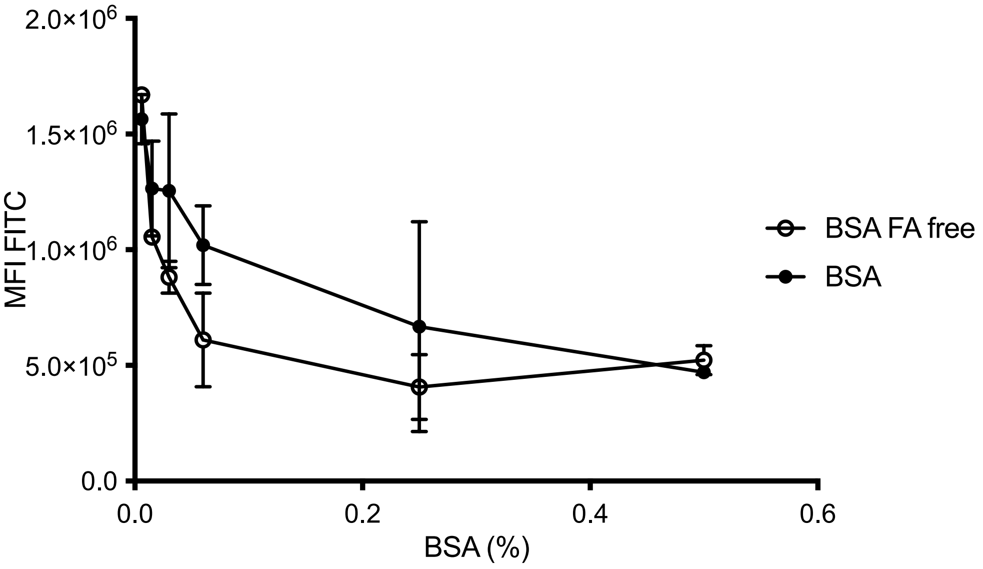

Figure S9: Uptake of Bdpy-ML in presence of increasing concentration of bovine serum albumin (BSA) or FA-free BSA in THP-1 cells. Fluorescence levels as measured by flow cytometry, reflecting Bdpy-ML incorporation in THP-1 after 24h of incubation. Data are means of triplicates +/- SD.

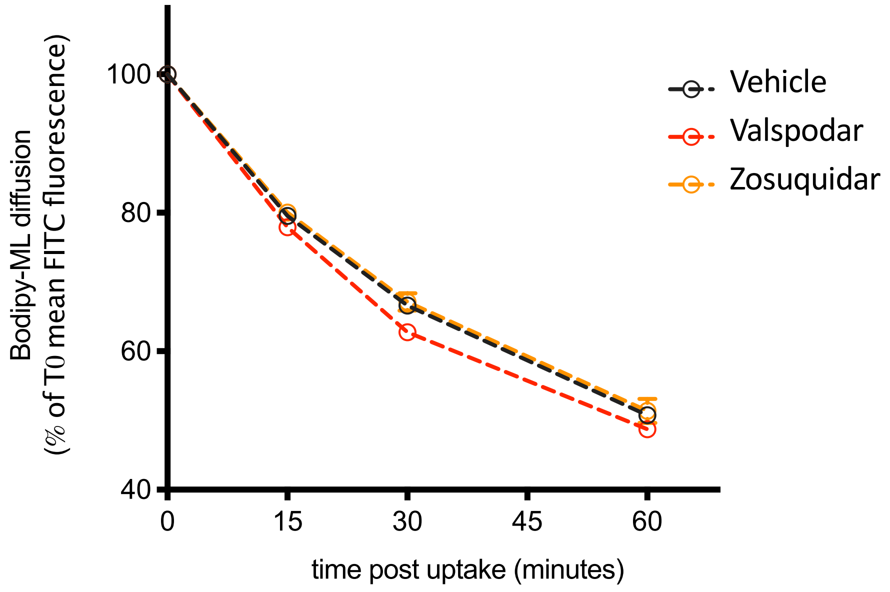

Figure S10: MDR1 is not involved in mycolactone detoxication. Kinetics of Bdpy-ML release in THP-1 cells in presence of vehicle or non-toxic doses of the MDR1 inhibitors valspodar (10µM) and zosuquidar (1µM). Data are expressed as mean percentages of triplicates to T0 and are representative of two independent experiments.
